# Low Temperature and Short-Term High-CO_2_ Treatment in Postharvest Storage of Table Grapes at Two Maturity Stages: Effects on Transcriptome Profiling

**DOI:** 10.3389/fpls.2016.01020

**Published:** 2016-07-13

**Authors:** Raquel Rosales, Irene Romero, Carlos Fernandez-Caballero, M. Isabel Escribano, Carmen Merodio, M. Teresa Sanchez-Ballesta

**Affiliations:** Departamento de Caracterización, Calidad y Seguridad, Instituto de Ciencia y Tecnología de Alimentos y Nutrición, ICTAN-CSIC, Ciudad UniversitariaMadrid, Spain

**Keywords:** *Vitis vinifera*, microarray analysis, carbon dioxide, low temperature, postharvest

## Abstract

Table grapes (*Vitis vinifera* cv. Cardinal) are highly perishable and their quality deteriorates during postharvest storage at low temperature mainly because of sensitivity to fungal decay and senescence of rachis. The application of a 3-day CO_2_ treatment (20 kPa CO_2_ + 20 kPa O_2_ + 60 kPa N_2_) at 0°C reduced total decay and retained fruit quality in early and late-harvested table grapes during postharvest storage. In order to study the transcriptional responsiveness of table grapes to low temperature and high CO_2_ levels in the first stage of storage and how the maturity stage affect these changes, we have performed a comparative large-scale transcriptional analysis using the custom-made GrapeGen GeneChip®. In the first stage of storage, low temperature led to a significantly intense change in grape skin transcriptome irrespective of fruit maturity, although there were different changes within each stage. In the case of CO_2_ treated samples, in comparison to fruit at time zero, only slight differences were observed. Functional enrichment analysis revealed that major modifications in the transcriptome profile of early- and late-harvested grapes stored at 0°C are linked to biotic and abiotic stress-responsive terms. However, in both cases there is a specific reprogramming of the transcriptome during the first stage of storage at 0°C in order to withstand the cold stress. Thus, genes involved in gluconeogenesis, photosynthesis, mRNA translation and lipid transport were up-regulated in the case of early-harvested grapes, and genes related to protein folding stability and intracellular membrane trafficking in late-harvested grapes. The beneficial effect of high CO_2_ treatment maintaining table grape quality seems to be an active process requiring the induction of several transcription factors and kinases in early-harvested grapes, and the activation of processes associated to the maintenance of energy in late-harvested grapes.

## Introduction

The maintenance and improvement of fruit quality during postharvest life is becoming increasingly important in response to a market where consumers demand products of high quality throughout the year. Low temperature storage is one of the most used methods to prolong postharvest quality and extend the shelf life of a broad range of horticultural commodities. Nevertheless, their use is sometimes limited depending on susceptibility to chilling injury and/or fungal decay. In addition to this, it is important to mention that fruit quality during cold storage is affected by the maturity stage of the fruit at harvest (Shin et al., 2008). Grapes (*Vitis vinifera* L.), which are a non-climacteric fruit with a relatively low rate of physiological activity, are not susceptible to injury at low (not freezing) temperature. For this reason, storage around 0°C is recommended for mature table grapes during postharvest. However, the storage life of grapes at low temperature is limited by their high susceptibility to fungal decay and sensitivity to serious water loss after harvest, which may result in stem drying and browning, and even shriveling of berries. The use of atmospheres modified in O_2_ and CO_2_ concentrations may extend the storage life of different fruit and vegetables at low temperature by reducing respiration, maintaining firmness and controlling decay. In this sense, we have shown that a 3-day treatment with high CO_2_ levels at 0°C is efficient in maintaining the quality of table grapes and controlling total decay (Romero et al., 2006; Sanchez-Ballesta et al., 2006). At transcriptional level, this gaseous treatment minimized or modified, the activation of cold-response mechanisms observed in non-treated grapes as a response to temperature shifts at 0°C (Sanchez-Ballesta et al., 2006; Fernandez-Caballero et al., 2012; Rosales et al., 2013).

The effect of high CO_2_ levels on the postharvest quality of small fruit and berries, such as grapes, depends on cultivar, maturity and storage length (Terry et al., 2009). Thus, Nunes et al. (2002) reported that three-quarters of colored strawberries responded better to controlled atmosphere storage at low temperature, maintaining greater firmness, better color, and reduced decay development than fully red fruit. In the case of table grapes, a delay in the harvest date did not affect to the effectiveness of the 3-day gaseous treatment. However, it was decisive in the increase of antioxidant activity by inducing anthocyanin accumulation in non-treated fruit stored at 0°C but not in CO_2_-treated ones (Romero et al., 2009). However, storage of early-harvested table grapes at 0°C in air causes a significant decrease in bound water levels and greater soluble-water K^+^ accumulation in comparison to CO_2_-treated, irrespective of the harvest year. Thus, some of the beneficial effects of the high CO_2_ treatment could be explained by restricting water mobility, and its influence on ion and volume homeostasis (Blanch et al., 2014).

With the development of microarray and next generation sequencing technology, global transcriptome analyses have been used to investigate the molecular regulatory networks underlying the responses of fruit during postharvest storage. Nevertheless, at the molecular level, very little is known about fruit response to short-term high-CO_2_ treatment applied during low temperature postharvest storage. Most previous works have been confined to the level of individual genes or small groups of genes. Thus, high CO_2_ effects were analyzed using microarrays in strawberries (Ponce-Valadez et al., 2009) and grape berries (Becatti et al., 2010). In the case of strawberries, the heterologous cDNA microarray TOM1 was used to compare gene expression differences between two cultivars treated with 20 kPa CO_2_ for 48 h. The cultivar Jewel accumulates acetaldehyde and ethanol in response to elevated CO_2_, whereas Cavendish does not accumulate these compounds under the same storage conditions. Despite the differences between both cultivars in terms of the number of genes differentially expressed, the distribution of their putative functions was very similar except for cDNAs with homology to genes encoding transcription factors and to genes involved in protein synthesis. Likewise, by using microarray, Becatti et al. (2010) reported that in detached wine grapes (cv. Trebbian, white skinned) a treatment with 30 kPa of CO_2_ for 3 days at 20°C was effective in altering the general metabolism, being functional categories related to protein and hormone metabolism, transport and stress highly represented in both skin and pulp tissues. Moreover, these authors also observed that the skin cells appeared to undergo more pronounced changes in transcriptome profiling in response to high CO_2_ than the pulp. Thus, fermentation, CHO metabolism, and redox regulation functional categories were represented only in the skin. To improve our understanding of the molecular mechanisms involved in the beneficial effect of short-term high-CO_2_ treatment on the quality of table grapes stored at low temperature, further transcriptomic studies are needed.

In this work, changes in the transcriptome of Cardinal table grape skin at different stages of maturity arising from exposure to high levels of CO_2_ and 0°C during 3 days were examined by using a custom made Affymetrix GrapeGen GeneChip™ (Lijavetzky et al., 2012). The results obtained improve our understanding of how table grapes respond to changes in atmospheric composition during the postharvest storage period which maintains their quality.

## Materials and methods

### Plant material

Table grapes (*Vitis vinifera* L. cv. Cardinal) were harvested from a commercial orchard in Sevilla (Spain) twice over 3 weeks. The first harvest began on the 13th June 2005 (early harvested, maturity index (MI) of 12.45 ± 0.01) and the second on the 5th July 2005 at commercial maturity (late harvested, MI of 41.08 ± 0.30). The field-packaged bunches were transported to the laboratory (time zero, t0) and those free from physical and pathological defects were randomly divided into two lots and stored up to 27 days at 0 ± 0.5°C and 95% relative humidity (RH) in two sealed neoprene containers of 1 m^3^ capacity. One lot of 6 replicate bunches was kept under normal atmosphere (non-treated fruit) and the other one, with same size, was kept under a gas mixture of 20 kPa CO_2_ + 20 kPa O_2_ + 60 kPa N_2_ (CO_2_-treated fruit). After 3 days, CO_2_-treated grapes were transferred to air under the same conditions as the non-treated fruit until the end of the storage period. At time 0 and after 3 days of storage under air or CO_2_ conditions, the skin of three biological replicates (each replicate consisting of 2 bunches) were collected, frozen in liquid nitrogen, ground to a fine powder and stored at −80°C until analysis. A scheme summarizing the experimental system is depicted in Supplementary Figure S1.

### Quality assessments

Berry quality assessment comprised soluble solids contents (SSC), titratable acidity (TA), pH and total decay. SSC was determined using a digital refractometer Atago PR-101 (Atago Co. Ltd., Japan) at 20°C and expressed in °Brix. TA was determined by titration with 0.1N NaOH up to pH 8.1 and results were expressed in % tartaric acid. The pH of the juice was measured in a pH meter with glass electrode.

Total decay was assessed on the basis of the total decay after removing and weighing the healthy berries. The weight of the decayed berries was calculated by subtracting healthy berries from the total cluster weight. Thus, total decay was expressed as the percentage of decayed berries with respect to the original cluster weight.

### Measurement of ion leakage

Membrane permeability was expressed by the relative ion leakage rate according to Lafuente et al. (1991) with slight modifications. Ten skin dishes (8 mm) were incubated in 10 mL of 0.3 M mannitol in Falcon 50-mL Conical Centrifuge Tubes. The tubes were shaken at 120 cycles per min and the conductivity of the solutions was measured after 1.5 h with a Crison 522 model conductivity meter. Tubes containing the mannitol solution and the tissue were heated to boiling for 15 min to permit complete leakage from the membranes. After cooling to room temperature with shaking, the total conductivity was measured. Ratios of ion leakage are expressed as percentage of the total conductivity per hour.

### RNA isolation and genechip® hybridization

In 2009, total RNA was extracted from skin samples according to the procedures described by Zeng and Yang (2002). RNA samples were treated with DNase I recombinant-RNase free (Roche) for removing possible genomic DNA contamination. Thereafter, final RNA purification was performed using RNeasy Mini Kit (Qiagen) following the manufacturer's instructions. Once RNA was purified, samples were analyzed at the Genomics Unit of the Spanish National Centre for Biotechnology (CNB-CSIC, Madrid). RNA integrity analyses were performed with an Agilent's Bioanalyzer 2100. A custom Affymetrix GrapeGen GeneChip® (Lijavetzky et al., 2012) containing 23,096 probe sets corresponding to 18,711 non-redundant transcripts was used. Probe synthesis, microarrays hybridization, washing, staining and scanning with the GeneChip™ Scanner 3000 were performed according to the Affymetrix GeneChip® Expression.

### Microarray data processing

Microarray data analyzed in this study have been submitted to the Gene Expression Omnibus database (www.ncbi.nlm.nih.gov/geo/) under the number GSE83275. Three biological replicates per experiment were processed to evaluate intra-specific variability. Signal values from all the microarray hybridizations were normalized together by applying the RMA (Robust Multiarray Average) algorithm using RMAExpress (Bolstad et al., 2003). Average of expression values for redundant probe sets was performed using GEPAS (Gene Expression Pattern Analysis Suite) software v4.0 (Herrero et al., 2003). Multivariate analysis as Principal Component Analysis (PCA) and Hierarchical Cluster Analysis (HCA) (ANOVA test, *P* < 0.01 after Bonferroni adjusted correction) over the normalized dataset was performed by using the MeV (MultiExperiment Viewer) TM4 Microarray Software Suite (Saeed et al., 2003).

### Microarray data analysis

SAM (Significance Analysis Microarray) algorithm implemented in TIGR MEV 4.5.1 (Saeed et al., 2003) was used to identify significantly altered gene expression in response to low temperature storage in air or high CO_2_ levels, and to generate false discovery rate (FDR) values for the analysis. SAM analyses were computed using two-class unpaired comparison on 1000 permutations. A FDR lower than 0.001 was applied in the analysis and only probe sets showing at least 1.5-fold change between conditions were considered as significant. Area-proportional Venn diagrams were generated in order to compare and visualize selected lists of genes by using BioVenn software (Hulsen et al., 2008). Probe set annotation was updated according to the 12X grape reference genome, V2 gene prediction (http://genomes.cribi.unipd.it/grape/; Vitulo et al., 2014).

The Database for Annotation, Visualization and Integrated Discovery (David 6.7; Huang et al. (2009), http://david.abcc.ncifcrf.gov) was used to classify specifically the genes with an altered expression either in air or high CO_2_ levels at 0°C, not taking into account those common to both cases, into functional groups based on Gene Ontology. Medium stringency was applied for the analyses. DAVID analysis identifies significantly enriched biological themes by examining for enrichment in over 40 different publicly available annotation categories, analyzing up- and down-regulated sets separately. A functional annotation chart (FACH) was generated by selecting the GOTERM_BP_FAT annotation category in DAVID, which allows enrichment analysis to highlight the most relevant Gene Ontology (GO) terms associated with a given gene list. The significance of gene enrichment in annotation terms was measured using the Expression Analysis Systematic Explorer (EASE) score, which is a modified Fisher Exact *P*-value, with application of the Benjamini-Hochberg method to correct for multiple comparisons, as applied in DAVID, with the threshold set at 0.05 (Huang et al., 2009). Functional Annotation Clustering (FAC) was then performed using the DAVID system. This higher level analysis combines single categories with a significant overlap in gene content and then assigns an enrichment score (ES; defined as the −log_10_ of the geometric mean of the unadjusted *p*-values for each single term in the cluster) to each cluster. In this instance, ES greater than 1.3 were considered significant. Fold-enrichment scores (FE) were also used to indicated the magnitude of enrichment for individual terms, and FE greater than 1.4 are suggestive of an informative change (Huang et al., 2009).

### Quantitative real-time RT-PCR (RT-qPCR)

Total RNA extraction was extracted as described above and treated with DNAse I recombinant-RNase free (Roche) to avoid DNA contamination. Then, 1 μg of RNA was used to synthesize cDNA by using the iScriptTM Reverse Transcription Supermix (Bio-Rad). Transcript levels were determined by RT-qPCR using a iCycler iQ thermal cycler (Bio-Rad) and SYBR Green dye (Bio-Rad). Gene-specific primers pairs for RT-qPCR (**Table 2**) were designed with Primer 3 software (Koressaar and Remm, 2007) using as templates the gene sequence from the *V. vinifera* 12X genomic sequence assembly Adam-Blondon et al., (2011; available at http://bioinfogp.cnb.csic.es/tools/GrapeGendb/). Each gene was evaluated at least in two independent runs. In order to calculate the efficiency of the reaction (optimal range 90–110%) and to establish the most suitable template concentration, cDNAs synthesized from serial dilutions (from 40 to 2.5 ng) of total RNA were amplified. Standard curves and linear equations were determined by plotting cycle threshold (Ct) values (y-axis) against logs of total RNA (x-axis). The efficiency of each individual run was calculated based on the raw fluorescence data (ΔRn) exported as output file and subsequently imported into the LinReg PCR program. The transcription levels were calculated relative to the calibrator sample (time 0) after normalization to the reference gene *actin1* from *V. vinifera*. Correlation coefficients (*r*^2^) between the log_2_-transformed expression values as measured by microarray and RT-qPCR were calculated. The specificity of products was validated by dissociation curve analysis and by agarose gel; and its sequences confirmed at the Genomic Department of the CIB-CSIC.

### Statistical analyses

Data were subjected to ANOVA (one-way analysis of variance) using the Fisher's Least Significant Difference (LSD) test to determinate the level of significance at *P* ≤ 0.05 (Statgraphics Centurion XVII, STSC, Rockville, MD).

## Results

### Effect of CO_2_ treatment and storage at 0°C on the quality of table grapes harvested at different stages of maturity

Irrespective of the maturity stage, treatment with high CO_2_ levels was effective in controlling total decay after 13 days at 0°C, the point at which the first symptoms of fungus infection appears (Table 1). However, as seen in Table 1, the effect of 3-day high CO_2_ levels on the quality parameters analyzed depended on the maturity stage. Thus, in the case of early-harvested grapes, the SSC content decreased in CO_2_-treated grapes, whereas the values of TA and pH did not change. However, in the case of non-treated grapes, values of SSC and TA decreased after 3 days at 0°C, with the pH values increasing. In late-harvested grapes, no changes were observed in SSC and TA values in any of the samples analyzed. By contrast, the pH values significantly decreased in both treated and non-treated samples, with the treated samples maintaining higher values.

**Table 1:** **Soluble solids content (SSC), titratable acidity (TA), pH, ion leakage total decay of early- and late-harvested table grapes cv. Cardinal treated with 20 kPa CO_**2**_ and stored during 13 days at 0°C**.

	**Early-harvested**			**Late-harvested**		
	**At harvest**	**13 days Air 0°C**	**3 days CO_2_ + 10 days 0°C**	**At harvest**	**13 days Air 0°C**	**3 days CO_2_ + 10 days 0°C**
Total decay	0a	10.16 ± 2.54c	4.34 ± 1.55b	0a	8.33 ± 0.9c	4.41 ± 1.82b
	**At harvest**	**3 days Air 0**°**C**	**3 days CO_2_ 0°C**	**At harvest**	**3 days Air 0**°**C**	**3 days CO_2_ 0°C**
Brix	11.90 ± 0b	11.03 ± 0.18a	11.15 ± 0.18a	15.73 ± 0.34b	16.28 ± 0.25b	14.28 ± 0.74b
TA (% tartaric acid)	0.96 ± 0b	0.86 ± 0.05a	0.92 ± 0.03ab	0.38 ± 0.01a	0.37 ± 0.02a	0.38 ± 0.01a
pH	3.23 ± 0a	3.32 ± 0.03b	3.26 ± 0.04a	4.15 ± 0.02c	3.78 ± 0.08a	3.90 ± 0.02b
Ion leakage (%)	52.77 ± 6.28a	62.31 ± 2.03b	54.09 ± 1.06ab	54.72 ± 0.17b	47.88 ± 1.05a	48.00 ± 4.86a

*Values are the mean of three replicate samples ±SE. Different letters within each row and harvest stage indicate that means are statistically different according to Fisher's protected LSD test (P ≤ 0.05)*.

Another difference between both maturity stages is the fact that late-harvested grapes showed a decrease in ion leakage in treated and non-treated samples, whereas a sharp increase was observed in the skin of non-treated early-harvested grapes in response to low temperature storage, which was avoided by the application of CO_2_ (Table 1).

### Main variation components in the grape skin transcriptome at two stages of maturity in response to 3-day high CO_2_ treatment and low temperature

**Figure 1:**
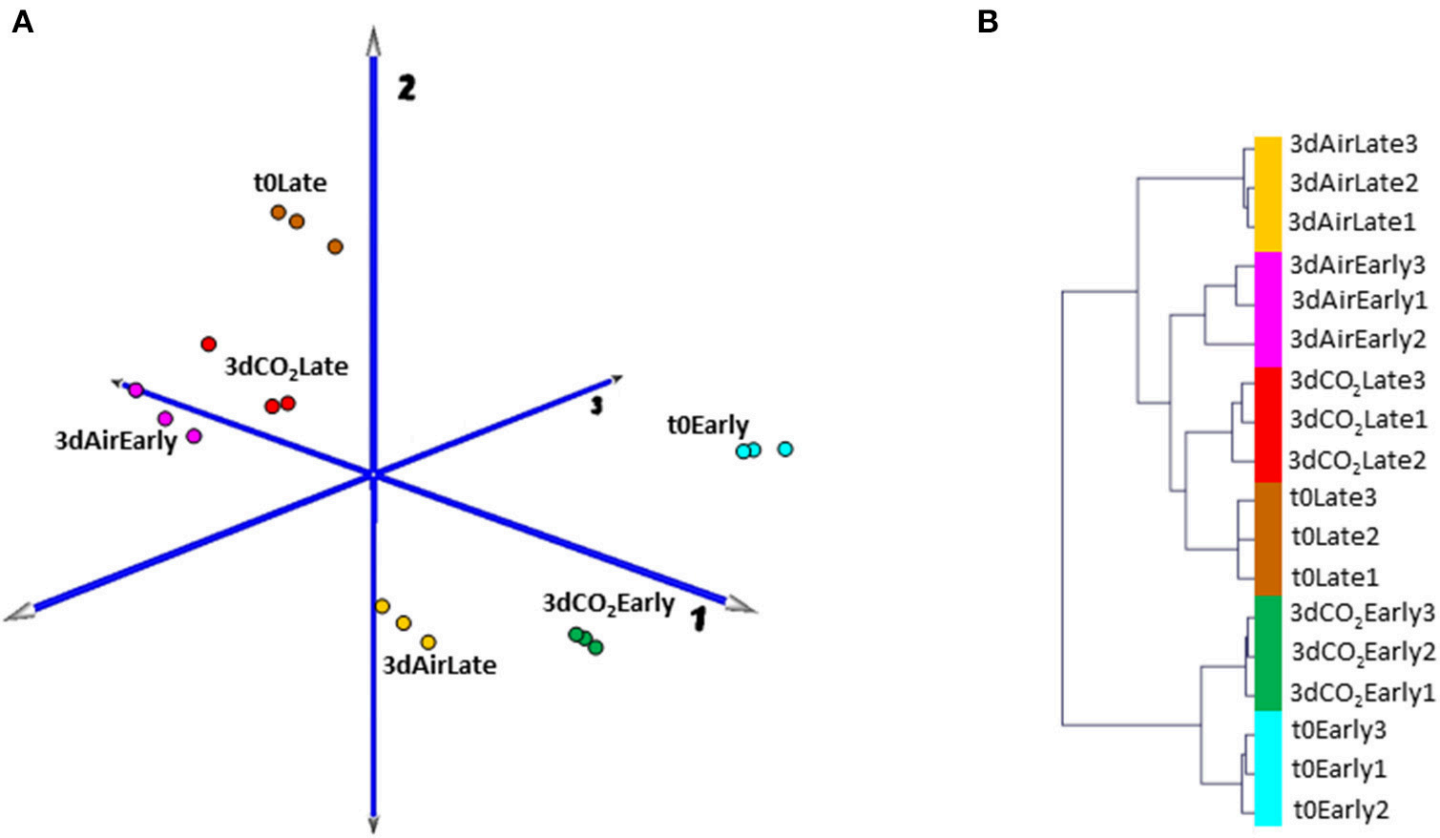
**(A)** Principal Component Analysis (PCA) and **(B)** Hierarchical Cluster Analysis (HCA) of transcriptome data from the skin of 3-day CO_2_-treated and non-treated grapes at early and late harvested stages. Colors in PCA for each condition are consistent with those in HCA. Three independent biological replicates of each condition were used and the analysis was carried out based on expression data previously RMA normalized between samples and after the average of expression values among redundant probe sets.

In order to obtain an overview of how low temperature and high CO_2_ levels affect the transcriptome of table grape skin in the first stage of storage and to analyze whether the maturity stage affects these changes we have used the GrapeGen GeneChip®. As a first approach to analyze the complexity of the gene expression dataset and to cluster samples according to their global gene expression profile, we performed a PCA and HCA (Figure 1) over the expression data of the eighteen analyzed samples, corresponding to three biological replicates at time zero, plus non-treated and CO_2_-treated samples at two maturity stages. Given that in all conditions, the transcriptional profiles of the three separate RNA replicate samples were tightly clustered, the experiment was considered highly reliable for further analysis. Principal component 1(PC1) explained 53% of the expression variation and separated samples in relation to the maturity stage (early vs. late harvested), with each CO_2_-treated sample close to its respective t0. However, non-treated fruit at both maturity stages clustered far from their respective samples at t0. PC2 accounted for 16% of the variance in the data set. Interestingly, samples from non-treated fruit harvested at early stage grouped near to samples from t0 and CO_2_-treated late harvested grapes, far from their respective t0 samples (variation on component 1), while late-harvested non-treated fruit clustered in the lower part of component 2 far from t0 and CO_2_-treated late harvested samples (Figure 1A). HCA confirmed results obtained by PCA, where t0 and 3-day CO_2_ treated samples at each maturity stage were clustered together but in independent branches, with samples from non-treated fruit at an early stage close to t0 and also to treated samples from late harvested fruit. Curiously, although 3-day non-treated samples at the late stage were in the same branch as their respective time zero, they were clustered into an independent group. These results indicated that 3 days of low temperature exposure led to an intense change in the grape skin transcriptome regardless of the fruit maturity stage but with different changes in each one, whereas slight differences were observed in CO_2_ treated samples in comparison with fruit at time zero (Figure 1B).

### Transcriptional bases for the response of grapes to low temperature and high levels of CO_2_ at two maturity stages

**Figure 2:**
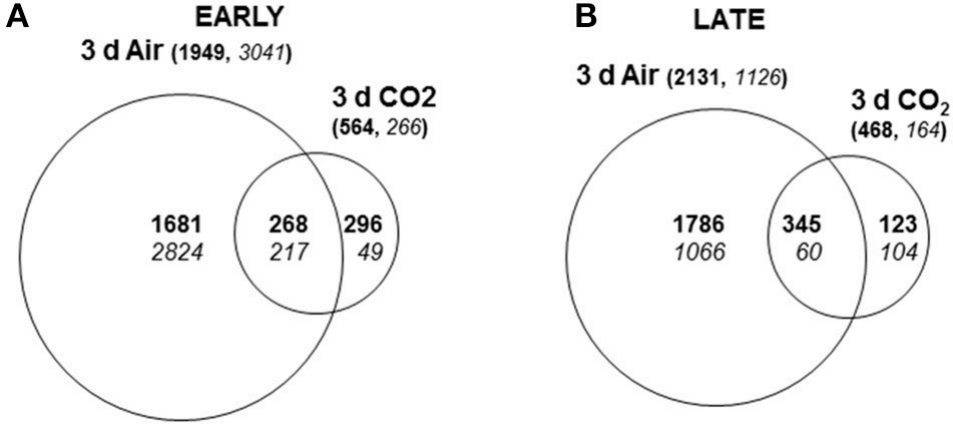
**Venn diagrams showing differentially expressed genes (SAM analysis, FDR < 0.001) in the skin of CO_**2**_-treated and non-treated grapes at the early and late maturity stage**. Expression levels for genes up-regulated (bold) and down-regulated (italics) in table grapes were compared to those of fruit at time zero at early **(A)** and late **(B)** maturity stages. Numbers in brackets are the sum of all induced or repressed genes in the early or late stage. The sizes of the circles are consistent with the total number of differentially expressed genes for each condition.

Venn diagrams summarize the number of overlapping differentially expressed genes (SAM analysis, FDR < 0.001, 1.5-fold change) in the skin of CO_2_-treated and non-treated early- or late-harvested table grapes with respect to each sample at t0 (Figures 2A,B). Major changes in the number of differentially expressed genes occurred in non-treated samples at early maturity stage where a total of 4990 non-redundant accumulated transcripts were identified, representing 26.6% of the total analyzed. Among the transcripts, 61% were down-regulated and 39% up-regulated by 3 days of storage at low temperature. Exposure of late-harvested table grapes at 0°C for 3 days also prompted major changes in gene expression (3257), with 2131 non-redundant transcripts (66%) up-regulated and 1126 (34%) down-regulated. The number of genes with a modified expression in response to the 3-day high CO_2_ treatment in comparison to t0 fruit was less remarkable than non-treated grapes at both maturity stages, although major changes also occurred in the early maturity stage (830) compared to the late-harvested one (632). In both cases, the number of up-regulated genes was higher than down-regulated ones.

In order to validate the microarray data, expression levels of 12 transcripts were analyzed by RT-qPCR using three biological replicates of t0, non-treated and CO_2_-treated samples at the two maturity stages (Table 2). Linear regression analyses displayed reliable *r*^2^ correlation coefficients between 0.73 and 0.99, confirming the validity of the microarray results.

**Table 2:** **Selected genes and primers used for quantitative RT-PCR and comparison between GrapeGen GeneChip® microarray and RT-qPCR gene expression data**.

**GrapegenDB 12Xv2 unique ID**	**Gene**	**Probe Set Annotation**	**Fw/Rv**	**Primer sequence 5′–3′**	***r*^2^**
VIT_208s0040g00470	CAM7	Calmodulin 7	F	CCAAGGAGCTAGGGACAGTG	0.9
			R	CTCGGAATGCTTCTTTCAGC	
VIT_204s0079g00690	GSTF11	Glutathione S-transferase F12	F	TCCTACCTCGAATGGGTGAG	0.9
			R	TTCGACAGCCTCTGCTCATA	
VIT_203s0038g02110	DNAJ	Chaperon protein DNAJ 11	F	GCAGCCTACTCCACCTTGTC	1
			R	ACCAGCACTGGTCAGTCTCC	
VIT_206s0004g08190	CRF4	ap2 erf domain-containing transcription factor	F	CCTCCTCCATTCCAACAAGA	0.9
			R	TCCCTCCACTCACCATTAGG	
VIT_209s0018g00240	WRKY40	Putative WRKY Transcription Factor 40	F	GAAGACGGGGAAGAAAAAGG	1
			R	CTTGGGTGGGTCAGTCAGAT	
VIT_202s0012g01040	NAC071	NAC domain-containing protein 71	F	CCATGGCTTATTGCAGGACT	1
			R	CAAATTCAACTTCCCCAGGA	
VIT_203s0088g00710	PRP1	Pathogenesis-related protein 1	F	GTGGTTCGCACATGCAACT	0.8
			R	CCTTTGTCAACTAAACGCACA	
VIT_200s0173g00170	RPS7	Ribosomal protein S7	F	CGATCCGTGAAAAAGATTCAA	1
			R	ATGAGTCGATCCGCCTACAC	
VIT_216s0013g01920		Kinase family protein	F	GTCACTTGATTTCTGTCCCAAT	0.8
			R	CCATTCATTATCCACATCCTCA	
VIT_211s0016g04080	MBF1c	Multiprotein-bridging factor 1c-like	F	CTTGCGAAGATGGAGAAGGT	0.8
			R	CGAGCGACGGACAAGACAC	
VIT_202s0025g00360	ACS6	1-aminocyclopropane-1-carboxylate synthase-like	F	GTTCCCATGGGTTTGCTTTA	0.9
			R	GTTGCATCATCCTCCATGTG	
VIT_213s0067g02130	ERD15	Dehydration-induced protein (ERD15)	F	GGAGGAGGAGAAGGAGCATC	0.7
			R	GAGCCTTCTCGAAGTGCCTA	

*Multiple linear regression analysis (r^2^) was performed for each reported gene including samples from all comparisons*.

### Functional analysis of the differential gene expression in non-treated grapes stored for 3 days at 0°C at both maturity stages

**Figure 3:**
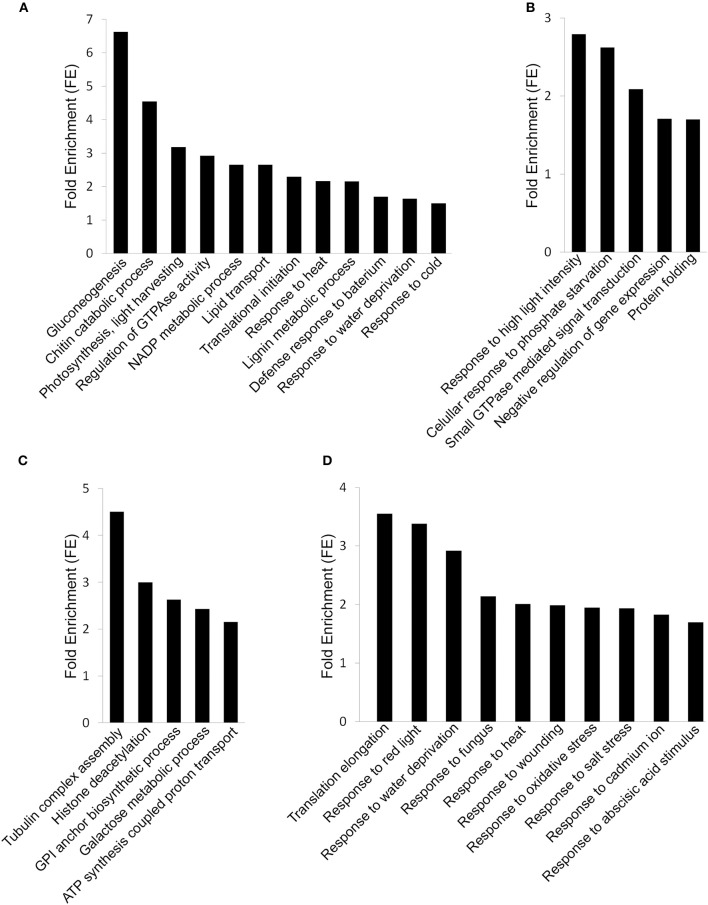
**DAVID Functional Annotation Chart (FACH) of normalized and annotated genes up- and down-regulated (fold change >1.5) in non-treated early- and late-harvested grapes stored for 3 days at 0°C. (A)** FACH of genes up-regulated in early-harvested grapes. **(B)** FACH of genes up-regulated in late-harvested grapes. **(C)** FACH of genes down-regulated in early-harvested grapes. **(D)** FACH of genes down-regulated in late-harvested grapes. Significance is determined by corresponding enrichment scores.

So as to understand the biological significance of the molecular changes underlying the specific effect of low temperature storage in non-treated grapes depending on the maturity stage, we performed a functional analysis on up- and down-regulated genes using DAVID. FACH provides data on the over-representation of GO category terms. At the early maturity stage, up-regulated transcriptional responses to low temperature storage overlapped broadly with response to abiotic (cold, heat and water deprivation) and biotic (defense response to bacterium) stimulus (*P* < 0.05 and fold-enrichment score (FE) between 2.17 and 1.50) (Figure 3A, Supplementary Table S1). Among the genes belonging to “response to heat” GO term, those coding for HSPs (heat shock proteins) were strongly represented. The genes implicated in cold tolerance such as *HOS15, LOS1, LOS4*, and temperature-induced lipocalin (*TIL*) related to the “response to cold” process were induced during the storage of non-treated early-harvested grapes at 0°C. In the case of up-regulated genes belonging to “response to water deprivation” process, these included genes coding for aquaporins, cysteine proteinases and dehydration-responsive protein. However, the most enriched up-regulated genes affected by low temperature were involved in the “gluconeogenesis” and “chitin catabolic” process (*P* < 0.05 and FE of 6.62 and 4.54, respectively). Thus, a phosphoenolpyruvate carboxykinase, a glucose-6-phosphate isomerase, two glyceraldehyde-3-phosphate dehydrogenases and four chitinases were induced (Figure 3A, Supplementary Table S1). Genes involved in “photosynthesis, light harvesting,” “regulation of GTPase activity,” and “NADP metabolic process” were also differentially expressed in response to low temperature storage (Figure 3A, Supplementary Table S1). These included genes encoding chlorophyll binding proteins, GTPases (ARF, RHO, and RAB) and fructose-bisphosphate aldolases. Another important over-represented GO terms were “lipid transport” (*P* < 0.05; *FE* = 2.65), including eight genes encoding lipid transfer proteins (LTPs), and “translational initiation” (*P* < 0.05; *FE* = 2.30), with ten genes coding for eukaryotic translation initiation factors (eIFs) (Figure 3A, Supplementary Table S1). Finally, within the “lignin metabolic process,” transcripts coding for laccases were over-represented (Figure 3A, Supplementary Table S1).

In late-harvested grapes, the transcriptional responses to low temperature were related to terms associated with stress such as “response to high light intensity” and “cellular response to phosphate starvation” (*P* < 0.05; *FE* = 2.79 and 2.62, respectively) (Figure 3B, Supplementary Table S2). Among the genes over-represented in these biological processes, we found genes coding for HSPs, which are different from those activated in early-harvested grapes, and also SPX (SYG1/Pho81/XPR1) domain proteins. Other transcripts up-regulated in late-harvested grapes stored at 0°C encoded calreticulin, chaperone DNAJ and peptidyl-prolyl isomerases, were associated to “protein folding” (*P* < 0.05; *FE* = 1.70) (Figure 3B, Supplementary Table S2). Cold storage also affected the expression of eight genes coding for Rab GTPases related to the “small GTPases meditated signal transduction” process (*FE* = 2.08).

The terms over-represented in down-regulated genes in response to low temperature depend on the maturity stage (Figures 3C,D, Supplementary Tables S1, S2). The most enriched down-regulated genes affected by low temperature in early-harvested grapes were involved in “tubulin complex assembly,” “histone deacetylation,” and “GPI anchor biosynthetic process” (*P* < 0.05; *FE* = 4.50, 3.00, and 2.62, respectively). Concerning energy metabolism, four aldose-1-epimerase related to “galactose metabolic process” (*P* < 0.05; *FE* = 2.42) were also down-regulated. Finally, the term “ATP synthesis coupled proton transport” (*P* < 0.05; *FE* = 2.15), including seven vacuolar (V-type) proton ATPases was over-represented in early-harvested grapes stored at 0°C (Figure 3C, Supplementary Table S1). By contrast, the analysis of the genes down-regulated by low temperature storage in late-harvested grapes mainly revealed terms which were related to response to abiotic and biotic stimulus (Figure 3D, Supplementary Table S2). Low temperature storage seems to affect protein biosynthesis due to the fact that genes associated with the most enriched GO term “translational elongation” (*P* < 0.05; *FE* = 3.55) were down-regulated in the skin of late-harvested grapes, including genes coding for two ribosomal proteins and two elongation factors (Figure 3D, Supplementary Table S2).

The FAC tool, FAC, clusters functionally related annotations into groups and ranks them according to importance by giving them enrichment score (ES). This tool showed four clusters of up-regulated genes and two clusters of down-regulated genes in non-treated early-harvested grapes, with significant ES ≥ 1.5 and *P* < 0.05 (Supplementary Table S3). Clusters of up-regulated genes were related to “photosynthesis,” “gluconeogenesis,” “defense response to bacterium,” and “NADP metabolic process” GO terms. Down-regulated genes were involved in clusters related to “ATP synthesis coupled proton transport” and “GPI anchor biosynthetic process.” In late-harvested grapes, the FAC of up-regulated genes were clustered into one group related to “cellular response to phosphate starvation.” There were two FAC down-regulated clusters in non-treated late-harvested grapes stored at 0°C. Genes were involved in “response to red light” (cluster 1) and “response to abscisic acid stimulus” (cluster 2) (Supplementary Table S3).

### Functional analysis of the differential gene expression in CO_2_-treated grapes stored for 3 days at 0°C at both maturity stages

**Figure 4:**
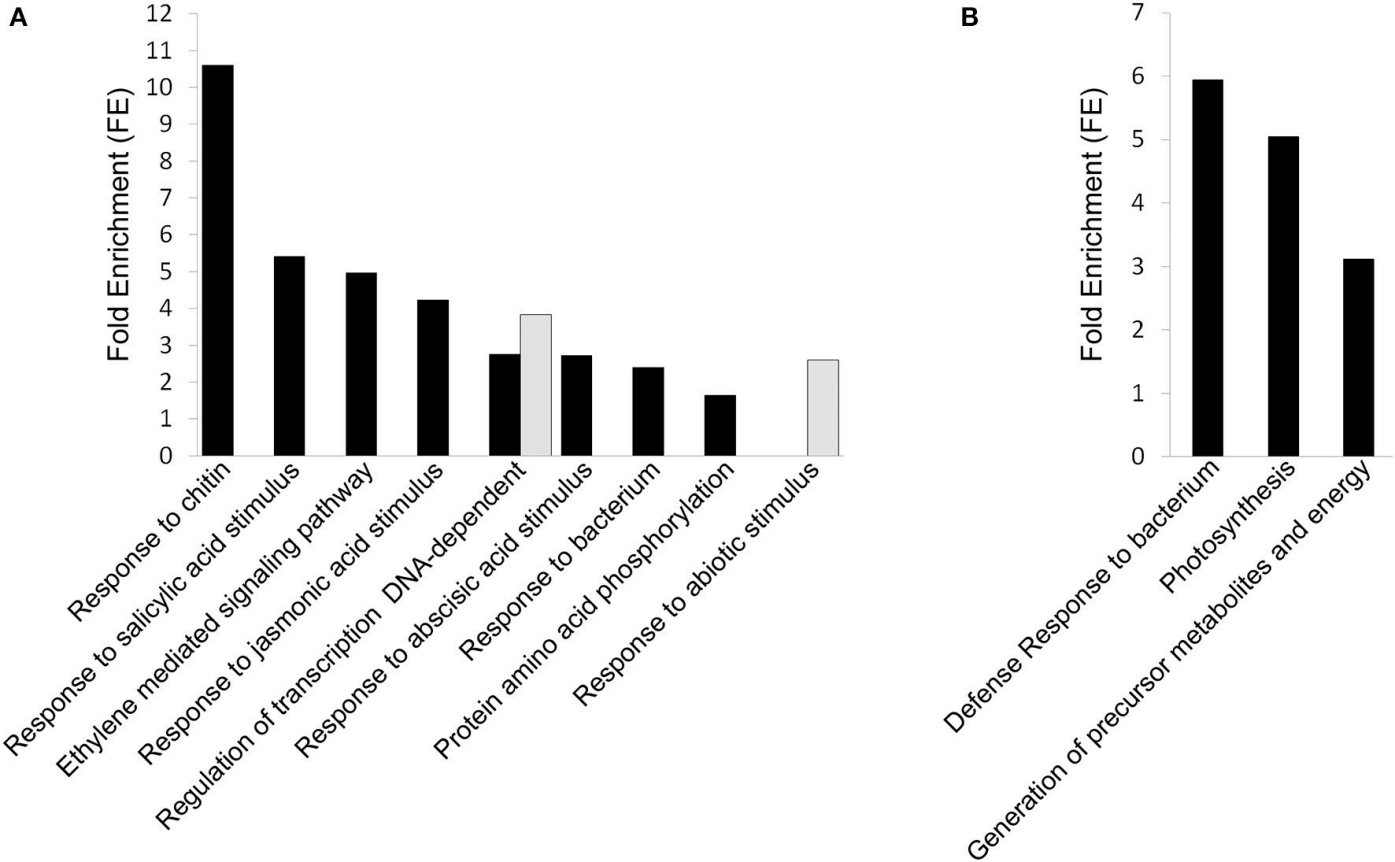
**DAVID Functional Annotation Chart (FACH) analysis of normalized and annotated genes up- and down-regulated (fold change >1.5) in 3-day CO_**2**_-treated early- and late- harvested grapes. (A)** FACH of genes up-regulated and down-regulated (>1.5-fold) in early-harvested grapes. **(B)** FACH of genes up-regulated in late-harvested grapes. Significance is determined by corresponding enrichment scores.

The application of high CO_2_ levels for 3 days at 0°C in early-harvested grapes significantly induced the expression of genes encoding mainly transcription factors associated with different GO terms such as “response to chitin,” “ethylene mediated signaling pathway,” “response to bacterium,” and “regulation of transcription, DNA-dependent” as well as response to salicylic, acid, jasmonic and abscisic acid stimulus (Figure 4A, Supplementary Table S4). Thus, we found twenty eight transcription factor genes (Table 3), including seven genes coding for ERF (Ethylene Responsive Factors) and five WRKYs. The gaseous treatment at 0°C also induced the expression of genes associated with “protein amino acid phosphorylation” GO term (*P* < 0.05; *FE* = 1.64), including fourteen genes encoding kinases mainly belonging to the receptor-like kinases (RLKs) family and cyclin-dependent kinase (Supplementary Table S4). However, in late-harvested grapes, only ten genes up-regulated by high CO_2_ levels were significantly associated with “defense response to bacterium,” “photosynthesis,” and “generation of precursor metabolites and energy” GO terms (Figure 4B, Supplementary Table S5).

**Table 3:** **Transcription factors up- and down-regulated in the skin of 3-day CO_**2**_-treated grapes at early stage**.

**GrapegenDB 12Xv2 unique ID**	**Probe set annotation**	**Fold change**
**UP-REGULATED**		
VIT_207s0141g00270	Auxin-induced protein 22D	1.81
VIT_207s0005g01450	bZIP transcription factor 53	3.91
VIT_212s0055g00420	bZIP transcription factor	1.48
VIT_206s0004g08190	Ethylene-responsive transcription factor CRF1	2.06
VIT_201s0011g03070	AP2/ERF and B3 domain-containing transcription factor RAV1-like	2.35
VIT_202s0234g00130	Ethylene responsive element binding factor 1A	2.93
VIT_207s0005g03230	Ethylene responsive element binding factor 1B	2.19
VIT_216s0013g01110	Ethylene-responsive transcription factor 4	4.36
VIT_216s0013g01120	Ethylene responsive element binding factor 5	1.60
VIT_200s0662g00040	Ethylene-responsive transcription factor ERF060	2.90
VIT_216s0013g00900	Ethylene-responsive transcription factor ERF105	1.90
VIT_207s0031g00220	Floral homeotic protein APETALA 2	1.51
VIT_208s0007g07550	GATA zinc finger	2.01
VIT_210s0003g01770	Heat stress transcription factor A-4a-like	1.63
VIT_213s0156g00260	Homeobox-leucine zipper protein HAT14-like	2.87
VIT_211s0016g04080	Multiprotein-bridging factor 1c	2.18
VIT_205s0049g01020	Myb transcription factor	2.25
VIT_203s0180g00210	Myb transcription factor 44	2.15
VIT_218s0001g11170	Myb transcription factor 73	2.85
VIT_200s0299g00060	Myb transcription factor 93	1.91
VIT_204s0008g05760	WRKY transcription factor 18	1.74
VIT_215s0046g02190	WRKY transcription factor 22	2.08
VIT_209s0018g00240	WRKY transcription factor 40	1.53
VIT_208s0040g03070	WRKY transcription factor 44	1.81
VIT_208s0058g01390	WRKY transcription factor 70	1.94
VIT_217s0000g01920	NF-X1-type zinc finger protein NFXL1-like	1.64
VIT_206s0004g04180	zinc finger protein ZAT11	2.67
VIT_218s0001g09230	Zinc-finger protein 1 ZAT10-like	1.86
**DOWN-REGULATED**		
VIT_213s0019g03550	Floral homeotic protein APETALA 2	−1.63
VIT_204s0008g06000	Ethylene-responsive transcription factor ERF003	−1.95
VIT_215s0048g02870	Homeobox-leucine zipper protein HB-7	−2.57
VIT_206s0004g02800	Homeodomain-leucine zipper protein Revoluta (REV)	−1.72
VIT_212s0142g00360	MAD-box transcripion factor	−1.77

The term “regulation of transcription, DNA-dependent” was also over-represented in genes down-regulated by high CO_2_ levels in early-harvested grapes (Figure 4A, Supplementary Table S4). This term included five transcription factors, belonging to the homeobox-leucine zipper, MAD-box, ERF, and AP2/EREBP families (Table 3). However, in the case of late-harvested grapes, no significant GO terms were over-represented in down-regulated genes by the gaseous treatment.

The FAC of genes up-regulated by high CO_2_ levels in early- and late-harvested grapes, revealed three clusters including GO categories of “response to chitin” and “regulation of transcription” (Cluster 1, *ES* = 5.79), “ethylene mediated signaling pathway” (Cluster 2; *ES* = 3.98) and “protein phosphorylation” (Cluster 3; *ES* = 1.81) (Supplementary Table S6). In late-harvested grapes, up-regulated ones were clustered into a group related to “defense response to bacterium” (*ES* = 2.58) (Supplementary Table S6).

## Discussion

To date, no studies have directly compared how low temperature and a short-term high-CO_2_ treatment affect the gene expression of table grapes harvested at two different maturity stages. In table grapes, minimum maturity requirements vary depending on cultivar, growing area and market. According to Codex Standards (codexstan255-2007) and the Economic Commission for Europe Standards (UNECE, 2010) for table grape maturity, the berries must be sufficiently developed and display satisfactory ripeness. In order to satisfy this requirement, the fruit must reach at least 16°Brix. Likewise, fruits with lower refractometric indices are accepted provided the sugar/acid ratio is at least equal to: (a) 20:1, if the Brix level is greater than or equal to 12.5° and less than 14°Brix, (b) 18:1, if the Brix level is greater than or equal to 14° and less than 16°Brix. In this work, maturity stages were selected to cover the effect of high CO_2_ levels on immature table grapes as well as on those meeting the level of maturity required.

It has been reported that in general, SSC, TA, and pH values remained quite constant in different varieties of grapes stored at 0°C under several conditions of controlled atmosphere (Artés-Hernández et al., 2006). However, our results indicated that the 3-day treatment with high CO_2_ levels affected the quality parameters analyzed depending on the maturity stage, although in both cases was effective controlling total decay. One of the common features accompanying chilling and senescence is increased membrane permeability, expressed as increasing leakage of ions which is used as an indicator of membrane damage. In Flame Seedless grapes, postharvest dip treatment with spermine (Champa et al., 2014) and preharvest salicylic acid application (Champa et al., 2015) extended cold storage postharvest and reduced the rate of membrane electrolyte leakage induced at low temperature. The significant increase in ion leakage observed in non-treated early-harvested grapes, in contrast with the values in CO_2_-treated fruit, seems to support our previous studies in which we reported that the 3-day gaseous treatment minimized or modified the activation of defense mechanisms that took place in non-treated grapes with a maturity stage near to the early-harvested grapes used in this study, as a response to temperature shifts at 0°C (Sanchez-Ballesta et al., 2006; Fernandez-Caballero et al., 2012; Rosales et al., 2013). Likewise, the differences observed between early- and late-harvested grapes could be related to the fact that the maturity stage affects the sensibility of fruit to develop chilling injury. Thus, chilling injury index in pre-yellow and yellow mango fruits was significantly lower than that of green fruits (Zhao et al., 2009b). Similarly, persimmon fruit (Salvador et al., 2005) and cucumber (Qian et al., 2013) were more susceptible to chilling injury at the earlier developmental stage.

As it is known, researchers have traditionally focused on transcriptional regulation as the main determinant of protein levels and, thus, cellular function. However, although the correlation between mRNA and protein abundance in the cell in a wide range of organisms is generally considered to be positive, it has been found to vary greatly between studies, cell types and organisms (reviewed by Plotkin, 2010). In our study, microarray analysis of the effect of low temperature and high CO_2_ levels on the skin of table grapes stored at two maturity stages, revealed substantial changes in the transcriptome as a response to storage at 0°C. By contrast, the proportion of genes which changed expression because of the 3-day treatment with high CO_2_ levels at 0°C was much lower at both maturity stages. These findings are in concordance with our previous works where the analysis of individual genes showed that the activation of cold-stress responses in the first stage of grape storage at 0°C in non-treated fruit, which was less noticeable in CO_2_-treated grapes (Sanchez-Ballesta et al., 2006; Rosales et al., 2013).

### Tolerance to low temperature of table grapes depends on maturity stage

The majority of transcripts up-regulated by low temperature in the skin of early-harvested grapes were related to GO terms associated with response to abiotic and biotic stress. Among them, the induction of *HOS15, LOS1, LOS4, TIL*, and *HSP* gene expression in response to low temperature storage could be related to the mechanisms activated in non-treated grapes to overcome low temperature storage. The expression of *HOS15*, a WD40-repeat protein, was induced by abiotic stresses including cold stress. Zhu et al. (2008) suggested that HOS15 functions as a repressor to control gene expression important to cold tolerance through chromatin modification. Likewise, the *los1* mutant of Arabidopsis, which is dysfunctional in the translational elongation factor 2, has reduced freeze tolerance and has an impaired ability to translate proteins at 0°C (Guo et al., 2002). Furthermore, LOS4, a DEAD-Box RNA helicase, also was found to be critically involved in chilling sensitivity of Arabidopsis (Gong et al., 2002). On the other hand, the up-regulation of *HSPs* was a common effect of low temperature storage on both early- and late-harvested grapes. The higher tolerance of different crops to chilling injury has been attributed to the production and accumulation of HSPs (Sanchez-Bel et al., 2012; reviewed by Aghdam et al., 2013). Yun et al. (2012) reported the induction of HSPs, cold regulated (COR) and temperature-induced lipocalins (TILs) gene expression in citrus fruit stored at low temperature, an observation which hints at the participation of these genes in promoting tolerance toward cold stress. In *V. vinifera*, a transcriptome analysis of berry ripening under high temperatures revealed the establishment of a thermotolerance response marked by the induction of twenty-one HSPs (Carbonell-Bejerano et al., 2013).

It is important to emphasize that “gluconeogenesis” and the “chitin catabolic process” showed the highest enrichment score in the FACH analysis of genes up-regulated by low temperature in the skin of early-harvested grapes. Gluconeogenesis is a fundamental metabolic process, allowing organisms to make sugars from non-carbohydrate stores such as lipids and protein. Phosphoenolpyruvate carboxykinase, which plays a pivotal role in gluconeogenesis by catalyzing the conversion of the C4 dicarboxylic acid oxaloacetic acid to phosphoenolpyruvate, has previously been associated with cold stress response (Xuan et al., 2013). Furthermore, although it is known that glyceraldehyde-3-phosphate dehydrogenases (GAPCs) act in glycolysis and gluconeogenesis, previous studies have demonstrated that they have a role to play in plant response to various stresses, such as cold, drought and hypoxia (reviewed by Kosová et al., 2011; Sanchez-Bel et al., 2012). In plants, GAPCs have been localized in the nucleus during cold stress (Bae et al., 2003), and their capacity to bind to DNA has been observed, in particular, to the coding sequence of the NADP-dependent malate dehydrogenase gene (Hameister et al., 2007). This fact seems to indicate that, in addition to its role in the gluconeogenesis and glycolysis, GAPCs may be involved in mediating stress signaling and signal transduction to the nucleus. On the other hand, it is important to note that the up-regulation of the four chitinases preceded the visual appearance of fungal attack in early-harvested grapes which took place after 13 days at 0°C. Furthermore, in a previous work we observed that expression levels of *chit1b* increased in the skin of table grapes after 3-day storage at 0°C and the overexpression of CHIT1b in *Escherichia coli* showed *in vitro* cryoprotective activity and retained catalytic activity at subzero temperature (Fernandez-Caballero et al., 2009), indicating a putative protective role during storage at low temperature.

In this study, the “lipid transport” process seems to play an important role in the response of early-harvested grapes at 0°C, inducing eight genes encoding LTPs. Transcript levels of LTPs increased in response to drought, salt and cold stresses (Jung et al., 2003). Stabilization of membranes, cuticle deposition and/or changes in cell wall organization, are believed to be responses to these stress factors. In peach fruit, Pavez et al. (2013) reported that expression of genes encoding antioxidant enzymes, HSPs and LTPs increased during cold storage and remained high after fruits were subjected to room temperature reconditioning. Likewise, the functions of this protein family as an enhancer of the phospholipids transfer between membranes and possibly as a molecule binding acyl chains are crucial in promoting tolerance to cold stress (Zhang et al., 2010).

On the other hand, environmental stress leads to the global reduction of protein synthesis, while the translation of selected stress responsive proteins can be maintained (Bavli-Kertselli et al., 2015). In these sense, our results showed the induction of ten genes coding eIFs in the skin of early-harvested grapes stored at 0°C. Translation initiation in eukaryotes depends on eIFs, with IF3 playing a central role in the polypeptide chain elongation interacting with many other translation initiation factors and being its expression induced by environmental stress (Kawaguchi and Bailey-Serres, 2002). Among the ten eIFs induced by low temperature, three eIF3 were identified, with IF3 considered the most important in terms of the selective translation of cold-shock mRNAs in *E. coli* (Giuliodori et al., 2004). In addition, a proteomic study indicated that banana fruit with down-regulated eIF5A showed the appearance of severe chilling injury, while the protein was significantly up-regulated in the ethylene-pretreated fruit without any chilling injury symptom (Li et al., 2015).

In reference to down-regulated genes by low temperature in the skin of early-harvested grapes, our results showed repression in the expression of five genes encoding tubulin folding cofactors. Microtubules constitute ubiquitous cytoskeletal elements required for a wide variety of cellular processes. They assemble from heterodimers consisting of α- and β- tubulins, and cofactors are required for the production of correctly folded and assembly-competent tubulin molecules (Gao et al., 1993). On winter wheat, Abdrakhamanova et al. (2003) showed that efficient cold acclimation was accompanied by an initial, partial disassembly of microtubules which was sufficient to trigger efficient cold acclimation. These authors suggest that microtubules, in addition to their role as effectors of the cold response, must have a function related to the efficient sensing of low temperature, culminating in the induction of the acclimation machinery. On the other hand, five genes coding for histone deacetylases were down-regulated in early-harvested grapes stored at 0°C. In maize, the expression of histone deacetylases was up-regulated during cold acclimation (Hu et al., 2011). HDA6 regulates the expression of several long-term cold stress-responsive genes and plays a role in the acquisition of freezing tolerance in plants (To et al., 2011).

Our results indicated the down-regulation of seven V-ATPase genes in early-harvested grapes stored at 0°C. One of the primary events of chilling injury in plants appears to be an inhibition of V-ATPase activity, which leads to an acidification of cytoplasm (Yoshida et al., 1999). Dietz et al. (2001) indicated that low-temperature induced a decline in V-ATPase activity and that the concomitant decrease in proton motive force could affect the solute compartmentation and possibly the hardiness of plants to low temperature. In this sense, we have observed that low temperature storage significantly increased the water-soluble K^+^ pool in the skin of table grapes, while high CO_2_ levels maintained it (Blanch et al., 2014). Likewise, in a proteomic study of cold acclimation of Arabidopsis, Minami et al. (2009) found many V-ATPases located in the microdomains which were down-regulated after chilling exposure, suggesting that these V-ATPases might contribute to cold or freezing tolerance by means of membrane trafficking regulation. Our data indicate that non-treated table grapes respond to low temperature by modulating, the expression of several genes to overtake cold stress together with other genes associated with the cellular stress which occurs during storage at a non-optimal temperature.

In the case of table grapes harvested at the optimal maturity standards, low temperature storage induced the expression of genes related to “response to phosphate starvation,” including *SPX2* and *SPX4*. At the molecular level, recent studies have shown that several proteins carrying the SPX domain are essential for maintaining phosphorus homeostasis in plants (reviewed by Secco et al., 2012). Likewise, constitutive overexpression of *OsSPX1* in tobacco plants resulted in decreased total leaf phosphorus concentration as well as the accumulation of free proline and sucrose, providing improved cold tolerance compared with the wild-type (Zhao et al., 2009a). In relation to stressful storage conditions, genes up-regulated in late-harvested grapes were also associated with the “protein folding” GO term. Protein folding stability is undoubtedly one of the most challenging problems in organisms undergoing stressful conditions. Thus, efficient protein repair systems and general protein stability facilitate survival under sudden changes in the environment. Among the genes involved in this term, it is interesting to note that in addition to chaperones DNAJ, genes encoding peptidyl-prolyl cis-trans isomerases (PPIases) were up-regulated. PPIases represent an important type of protein foldase, catalyzing the reversible conversion of peptidyl prolyl bond from *cis* to *trans* which is a rate-limiting step in folding proteins (Fischer and Schmid, 1999). Four structurally distinct subfamilies of PPIases (cyclophilins, FKBP, parvulins, and PP2A phosphatase activators) were characterized (Lu et al., 2007). Our results showed that genes coding for different cyclophilins and FKBP subfamilies were up-regulated by low temperature in late-harvested grapes, suggesting that a larger number of PPIases are required in order to facilitate the efficient folding of proteins during storage. Different members of cyclophilin subfamily were directly linked to multiple stresses. In this sense, Budiman et al. (2011) proposed that foldase and chaperone activities of PPIases are the main requirements with which to overcome the cold-stress problem in microorganisms caused by protein folding.

It is important to note that the number of genes repressed in late-harvested grapes stored at 0°C was almost three times lower than those of early-harvested grapes. Among them, genes related to the response to biotic and abiotic stress together with the GO term “translation elongation” (60 s ribosomal protein and elongation factors) were affected. Earlier studies have shown that the translational machinery can be modified by thermal stress. Heat-stressed seedlings of *Brassica napus* have been shown to have low levels of translation elongation factors (Dhaubhadel et al., 2002). Likewise, whereas the EF-1α mRNA levels increased at low temperature in maize leaves, time-course experiments over 24 h at 5°C showed that in the case of roots, the overall mRNA level of EF-lα was transiently decreased (Berberich et al., 1995).

### Role of 3-day high CO_2_ levels in table grape response to low temperature

The transcriptomic analysis showed that both early- and late-harvested grapes presented more up-regulated genes than down-regulated ones as a response to high CO_2_ levels at 0°C. However, Becatti et al. (2010) observed that in the case of detached wine grapes, the application of high CO_2_ levels (30%) for 3 days at 20°C induced the repression of more genes in the skin and pulp compared to those activated. Similar results were obtained in a RNA-Seq analysis performed with nectarines stored under controlled atmospheres (15% CO_2_) for 21 days at 4°C (Sanhueza et al., 2015).

The application of a 3-day treatment with high CO_2_ levels at 0°C in early-harvested grapes significantly induced the expression of twenty eight transcription factors related to the GO terms “response to chitin,” “ethylene mediated signaling pathway,” and “regulation of transcription, DNA-dependent.” Because stress gene induction occurs primarily at the level of transcription, transcription factors interact with *cis*-elements in the promoter regions of several stress-related genes and thus up-regulate the expression of many downstream genes resulting in imparting abiotic stress tolerance (Agarwal and Jha, 2010). Plants devote a large portion of their genome capacity to transcription, with the *V. vinifera* genome coding in excess of 1200 transcription factors classified into 58 families (Grimplet et al., 2012; Jin et al., 2014). In this study, we found the induction of transcription factor genes belonging to different families such as ERF (Ethylene Responsive Factors), a subfamily of the APETALA2 (AP2)/ERF transcription factor family, as well to the WRKY, MYB, basic-domain leucine-zipper (bZIP), heat stress transcription factor and zinc finger. Expression data from different plants species, including *V. vinifera*, have indicated that different members of the transcription factors participate in plant responses to cold stress. After 4 h of cold treatment, a total of 70 up-regulated and 18 down-regulated transcription factors were identified in leaves of *V. amurensis*, a wild grapevine species with remarkable cold-tolerance, while 68 up-regulated and 43 down-regulated transcription factors were identified in *V. vinifera* (Xin et al., 2013). In shoot apices of *V. vinifera* cv. Muscat Hamburg, Wang et al. (2014) observed the induction of fifteen *VvWRKYs* in response to cold stress. To our knowledge, however, there is no information available about the participation of transcription factors on the response of fruit to high CO_2_ levels and low temperature. In this sense, we observed that the application of 3-day high CO_2_ levels at 0°C induced the expression of *CBF1* and *CBF4* in the pulp of table grapes and *CBF4* in the rachis (Fernandez-Caballero et al., 2012). The fact that CBFs also belong to the AP2/ERF transcription factor family seems to indicate that this family plays a prominent role in the beneficial effect of the gaseous treatment in table grapes. Likewise, the thermotolerance response of Muscat Hamburg berries ripened at high temperature was coincident with the up-regulation of *ERF* transcription factors, suggesting their participation in the maintenance of the acclimation response (Carbonell-Bejerano et al., 2013).

In early-harvested grapes, the gaseous treatment also induced genes that encoded kinases belonging mainly to the RLKs family. Phosphorylation mediated by kinases is one of the most ordinary mechanisms by which environmental cues are transduced and protein function is regulated in eukaryotic cells. It is known that RLKs mediate cold acclimation. Thus, a null mutant for a gene encoding a plasma-membrane RLK, showed a reduction in cold-induced gene expression as well as in the capacity to cold acclimate (Yang et al., 2010). Dehydrins are also proteins involved in cold acclimation and are post-translationally modified by phosphorylation. They accumulate in response to diverse abiotic stresses, including low temperature, and act as molecular chaperones (Hara, 2010). In a recent work, we observed that high CO_2_ levels induced the accumulation of DHN44 in the skin of table grapes and *in vitro* assays indicated that this dehydrin can be phosphorylated (Navarro et al., 2015).

In the early stage, the most enriched down-regulated genes in CO_2_-treated grapes codified for five transcription factors belonging to the homeobox-leucine zipper, MAD-box and ERF families. This finding, together with the fact that transcription factors are mainly up-regulated in treated grapes, seems to indicate that the gaseous treatment could be an active process requiring the regulation of transcription factors.

In late-harvested grapes, only ten genes up-regulated by high CO_2_ levels at 0°C were related to the GO terms “defense response,” “photosynthesis,” and “generation of precursor metabolites and energy.” Proteomic analyses indicated that tolerant plants can induce several protective mechanisms more efficiently than sensitive ones because they are able to maintain sufficient rates of several metabolic processes, especially those associated with energy metabolism under adverse stress conditions (Ingle et al., 2005; Dumont et al., 2011). Maintaining sufficient rates of processes associated with energy metabolism is extremely important for an efficient stress acclimation since it is an active process associated with *de novo* biosynthesis of several stress-protective compounds (Bartels and Sunkar, 2005). Finally, as we have already mentioned, the gaseous treatment was effective controlling total decay in table grapes at both maturity stages. Curiously, in early- and late-harvested grapes the GO term “defense to bacterium” was over-represented although different genes up-regulated were included in each case.

## Conclusions

The transcriptional responses of table grapes to low temperature and high CO_2_ levels depend on the stage of maturity. While it is true that cold storage had similar effect on fruit quality regardless of the date of harvest and that high CO_2_ treatment was effective in controlling total decay and maintaining quality at the end of the storage period in both maturity stages (data not shown), our results indicate that they do so through different mechanisms and the modifications in the transcriptome profile seem to be different. Major modifications in the transcriptome profile of early- and late-harvested grapes storage at 0°C are linked to biotic and abiotic stress-responsive terms. However, specific transcriptional modifications were observed depending on the date of harvest. The results obtained in this study indicate that in both cases there is a reprogramming of the transcriptome during the first stage of storage at a non-optimal temperature. Thus, in early-harvested grapes changes associated with gluconeogenesis, photosynthesis, mRNA translation and lipid transport occurs, whereas the maintenance of protein folding stability and intracellular membrane trafficking seems to play an important role in late-harvested grapes. Similarly, the cellular response to low temperature stress seems to be related to maintaining the ion homeostasis, as reflected in the inhibition of key transport proteins such as V-ATPases in early-harvested grapes, which are thought to participate in the very early response to cold stress by regulating membrane trafficking.

In order to maintain early-harvested table grape quality, the high CO_2_ treatment seems to be an active process requiring the activation of transcription factors, as well as protein kinases implicated in the regulation of protein function. Likewise, although the number of genes significantly associated with GO-terms in late-harvested grapes under high CO_2_ levels was low, there seems to be an active process associated with maintaining the energy of the fruit.

## Author contributions

RR, RNA extraction, RT-qPCR analysis and improved the manuscript. IR, table grape quality assessments, edited and improved the first draft of the manuscript. CF, RNA extraction and table grape quality assessments. ME and CM, improved the manuscript. MS, designed the research, data analysis and prepared the first draft of the manuscript. All authors have read and approved this manuscript.

### Conflict of interest statement

The authors declare that the research was conducted in the absence of any commercial or financial relationships that could be construed as a potential conflict of interest.

## References

[B1] Abdrakhamanova A. Wang Q. Y. Khokhlova L. Nick P. (2003). Is microtubule disassembly a trigger for cold acclimation? Plant Cell Physiol. 44, 676–686. 10.1093/pcp/pcg097 12881495

[B2] Adam-Blondon A.-F. Jaillon O. Vezzulli S. Zharkikh A. Troggio M. Velasco R. (2011). Genome sequence initiatives in Genetics, Genomics and Breeding of Grapes, eds Adam-Blondon A.-F. Martinez-Zapater J. M. Kole C. (Enfield, NH: Science Publishers), 211–234.

[B3] Agarwal P. K. Jha B. (2010). Transcription factors in plants and ABA dependent and independent abiotic stress signaling. Biol. Plantarum 54, 201–212. 10.1007/s10535-010-0038-7

[B4] Aghdam M. S. Sevillano L. Flores F. B. Bodbodak S. (2013). Heat shock proteins as biochemical markers for postharvest chilling stress in fruits and vegetables. Sci. Hortic. 160, 53–64. 10.1016/j.scienta.2013.05.020

[B5] Artés-Hernández F. Tomàs-Barberán F. A. Artés F. (2006). Modified atmosphere packaging preserves quality of SO_2_-free ‘Superior seedless’ table grapes. Postharvest Biol. Technol. 39, 146–154. 10.1016/j.postharvbio.2005.10.006

[B6] Bae M. S. Cho E. J. Choi E. Y. Park O. K. (2003). Analysis of the *Arabidopsis* nuclear proteome and its response to cold stress. Plant J. 36. 652–663. 10.1046/j.1365-313X.2003.01907.x 14617066

[B7] Bartels D. Sunkar R. (2005). Drought and salt tolerance in plants. Crit. Rev. Plant Sci. 24, 23–58. 10.1080/07352680590910410

[B8] Bavli-Kertselli I. Melamed D. Bar-Ziv L. Volf H. Arava Y. (2015). Overexpression of eukaryotic initiation factor 5 rescues the translational defect of *tpk1*^*w*^ in a manner that necessitates a novel phosphorylation site. FEBS J. 282, 504–520. 10.1111/febs.13158 25417541

[B9] Becatti E. Chkaiban L. Tonutti P. Forcato C. Bonghi C. Ranieri A. (2010). Short term postharvest carbon dioxide treatments induce selective molecular and metabolic changes in grape berries. J. Agric. Food Chem. 58, 8012–8020. 10.1021/jf100936x 20557098

[B10] Berberich T. Sugawara K. Harada M. Kusano T. (1995). Molecular cloning, characterization and expression of an elongation factor 1α gene in maize. Plant Mol. Biol. 29, 611–615. 10.1007/BF00020988 8534856

[B11] Blanch M. Fernandez-Caballero C. Sanchez-Ballesta M. T. Escribano M. I. Merodio C. (2014). Accumulation and distribution of potassium and its association with water balance in the skin of Cardinal table grapes during storage. Sci. Hort. 175, 223–228. 10.1016/j.scienta.2014.06.016

[B12] Bolstad B. M. Irizarry R. A. Astrand M. Speed T. P. (2003). A comparison of mormalization methods for high density oligonucleotide array data based on bias and variance. Bioinformatics 19, 185–193. 10.1093/bioinformatics/19.2.185 12538238

[B13] Budiman C. Koga Y. Takano K. Kanaya S. (2011). FK506-Binding Protein 22 from psychrophilic bacterium, a cold shock-inducible peptidyl prolyl isomerase with the ability to assist in protein folding. Int. J. Mol. Sci. 12, 5261–5284. 10.3390/ijms12085261 21954357 PMC3179164

[B14] Carbonell-Bejerano P. Santa María E. Torres-Pérez R. Royo C. Lijavetzky D. Bravo G.. (2013). Thermotolerance responses in ripening berries of *Vitis vinifera* L. cv Muscat Hamburg. Plant Cell Physiol. 54, 1200–1216. 10.1093/pcp/pct071 23659918

[B15] Champa W. A. H. Gill M. I. Mahajan B. V. Arora N. K. (2015). Preharvest salicylic acid treatments to improve quality and postharvest life of table grapes (*Vitis vinifera* L.) cv. Flame Seedless. J. Food Sci. Technol. 52, 3607–3616. 10.1007/s13197-014-1422-7 26028743 PMC4444924

[B16] Champa W. A. H. Gill M. I. S. Mahajan B. V. C. Arora N. K. (2014). Postharvest treatment of polyamines maintains quality and extends shelf-life of table grapes (*Vitis vinifera* L.) cv. Flame Seedless. Postharvest Biol. Technol. 91, 57–63. 10.1016/j.postharvbio.2013.12.014

[B17] Dhaubhadel S. Browning K. S. Gallie D. R. Krishna P. (2002). Brassinosteroid functions to protect the translational machinery and heat-shock protein synthesis following thermal stress. Plant J. 29, 681–691. 10.1046/j.1365-313X.2002.01257.x 12148527

[B18] Dietz K. J. Tavakoli N. Kluge C. Mimura T. Sharma S. S. Harris G. C.. (2001). Significance of the V-type ATPase for the adaptation to stressful growth conditions and its regulation on the molecular and biochemical level. J. Exp. Bot. 52, 1969–1980. 10.1093/jexbot/52.363.1969 11559732

[B19] Dumont E. Bahrman N. Goulas E. Valot B. Sellier H. Hilbert J. L.. (2011). A proteomic approach to decipher chilling response from cold acclimation in pea (*Pisum sativum* L.). Plant Sci. 180, 86–98. 10.1016/j.plantsci.2010.09.006 21421351

[B20] Fernandez-Caballero C. Romero I. Goñi O. Escribano M. I. Merodio C. Sanchez-Ballesta M. T. (2009). Characterization of an antifungal and cryoprotective class I chitinase from table grape berries (*Vitis vinifera* Cv. Cardinal). J. Agric. Food Chem. 57, 8893–8900. 10.1021/jf9016543 19769368

[B21] Fernandez-Caballero C. Rosales R. Romero I. Escribano M. I. Merodio C. Sanchez-Ballesta M. T. (2012). Unraveling the roles of *CBF1, CBF4* and dehydrin 1 genes in the response of table grapes to high CO_2_ levels and low temperature. J. Plant Physiol. 169, 744–748. 10.1016/j.jplph.2011.12.018 22341570

[B22] Fischer G. Schmid F. X. (1999). Peptidyl-prolyl cis/trans isomerases in Molecular Chaperones and Folding Catalysis: Regulation, Cellular Function, and Mechanisms, ed Bukau B. (Amsterdam: Harwood Academic Publishers), 461–489.

[B23] Gao Y. Vainberg I. E. Chow R. L. Cowan N. J. (1993). Two cofactors and cytoplasmic chaperonin are required for the folding of α- and β-tubulin. Mol. Cell. Biol. 13, 2478–2485. 10.1128/MCB.13.4.2478 8096061 PMC359568

[B24] Giuliodori A. M. Brandi A. Gualerzi C. O. Pon C. L. (2004). Preferential translation of cold-shock mRNAs during cold adaptation. RNA 10, 265–276. 10.1261/rna.5164904 14730025 PMC1370538

[B25] Gong Z. Lee H. Xiong L. Jagendorf A. Stevenson B. Zhu J. K. (2002). RNA helicase-like protein as an early regulator of transcription factors for plant chilling and freezing tolerance. Proc. Natl. Acad. Sci. U.S.A. 99, 11507–11512. 10.1073/pnas.172399299 12165572 PMC123286

[B26] Grimplet J. Van Hemert J. Carbonell-Bejerano P. Díaz-Riquelme J. Dickerson J. Fennell A.. (2012). Comparative analysis of grapevine whole-genome gene predictions, functional annotation, categorization and integration of the predicted gene sequences. BMC Res. Notes 5:213. 10.1186/1756-0500-5-213 22554261 PMC3419625

[B27] Guo Y. Xiong L. Ishitani M. Zhu J. K. (2002). An *Arabidopsis* mutation in translation elongation factor 2 causes superinduction of *CBF/DREB1* transcription factor genes but blocks the induction of their downstream targets under low temperatures. Proc. Natl. Acad. Sci. U.S.A. 99, 7786–7791. 10.1073/pnas.112040099 12032361 PMC124352

[B28] Hameister S. Becker B. Holtgrefe S. Strodtköetter I. Linke V. Backhausen J. E.. (2007). Transcriptional regulation of NADP-dependent malate dehydrogenase: comparative genetics and identification of DNA-binding proteins. J. Mol. Evol. 65, 437–455. 10.1007/s00239-007-9025-9 17925997

[B29] Hara M. (2010). The multifunctionality of dehydrins: an overview. Plant Signal. Behav. 5, 503–508. 10.4161/psb.11085 20139737 PMC7080494

[B30] Herrero J. Al-Shahrour F. Díaz-Uriarte R. Mateos A. Vaquerizas J. M. Santoyo J.. (2003). GEPAS: a web-based resource for microarray gene expression data analysis. Nucleic Acids Res. 31, 3461–3467. 10.1093/nar/gkg591 12824345 PMC168997

[B31] Hu Y. Zhang L. Zhao L. Li J. He S. Zhou K.. (2011). Trichostatin A selectively suppresses the cold-induced transcription of the *ZmDREB1* gene in maize. PLoS ONE 6:22132. 10.1371/journal.pone.0022132 21811564 PMC3141014

[B32] Huang D. W. Sherman B. T. Lempicki R. A. (2009). Systematic and integrative analysis of large gene lists using DAVID bioinformatics resources. Nat. Protoc. 4, 44–57. 10.1038/nprot.2008.211 19131956

[B33] Hulsen T. de Vlieg J. Alkema W. (2008). BioVenn–a web application for the comparison and visualization of biological lists using area-proportional Venn diagrams. BMC Genomics 9:488. 10.1186/1471-2164-9-488 18925949 PMC2584113

[B34] Ingle R. A. Smith J. A. C. Sweetlove L. J. (2005). Responses to nickel in the proteome of the hyperaccumulator plant *Alyssum lesbiacum*. Biometals 18, 627–641. 10.1007/s10534-005-2999-0 16388402

[B35] Jin J. P. Zhang H. Kong L. Gao G. Luo J. C. (2014). PlantTFDB 3.0: a portal for the functional and evolutionary study of plant transcription factors. Nucleic Acids Res. 42, 1182–1187. 10.1093/nar/gkt1016 24174544 PMC3965000

[B36] Jung H. W. Kim W. Hwang B. K. (2003). Three pathogen-inducible genes encoding lipid transfer protein from pepper are differentially activated by pathogens, abiotic, and environmental stresses. Plant Cell Environ. 26, 915–928. 10.1046/j.1365-3040.2003.01024.x 12803619

[B37] Kawaguchi R. Bailey-Serres J. (2002). Regulation of translational initiation in plants. Curr. Opin. Plant Biol. 5, 460–465. 10.1016/S1369-5266(02)00290-X 12183186

[B38] Koressaar T. Remm M. (2007). Enhancements and modifications of primer design program Primer3. Bioinformatics 23, 1289–1291. 10.1093/bioinformatics/btm091 17379693

[B39] Kosová K. Vítámvás P. Prášil I. T. Renaut J. (2011). Plant proteome changes under abiotic stress-contribution of proteomics studies to understanding plant stress response. J. Proteomics 74, 1301–1313. 10.1016/j.jprot.2011.02.006 21329772

[B40] Lafuente M. T. Belver A. Guye M. G. Salveit M. E. (1991). Effect of temperature conditioning on chilling injury of cucumber cotyledons. Possible role of abscisic and heat shock proteins. Plant Physiol. 95, 443–449. 10.1104/pp.95.2.443 16668003 PMC1077550

[B41] Li T. Yun Z. Zhang D. Yang C. Zhu H. Jiangm Y.. (2015). Proteomic analysis of differentially expressed proteins involved in ethylene-induced chilling tolerance in harvested banana fruit. Front. Plant Sci. 6:845. 10.3389/fpls.2015.00845 26528309 PMC4606070

[B42] Lijavetzky D. Carbonell-Bejerano P. Grimplet J. Bravo G. Flores P. Fenoll J.. (2012). Berry flesh and skin ripening features in *Vitis vinife*ra as assessed by transcriptional profiling. PLoS ONE 7:39547. 10.1371/journal.pone.0039547 22768087 PMC3386993

[B43] Lu K. P. Finn G. Lee T. H. Nicholson L. K. (2007). Prolyl *cis*-*trans* isomerization as a molecular timer. Nat. Chem. Biol. 3, 619–629. 10.1038/nchembio.2007.35 17876319

[B44] Minami A. Fujiwara M. Furuto A. Fukao Y. Yamashita T. Kamo M.. (2009). Alterations in detergent-resistant plasma membrane microdomains in *Arabidopsis thaliana* during cold acclimation. Plant Cell Physiol. 50, 341–359. 10.1093/pcp/pcn202 19106119

[B45] Navarro S. Vazquez-Hernandez M. Rosales R. Sanchez-Ballesta M. T. Merodio C. Escribano M. I. (2015). Differential regulation of dehydrin expression and trehalose levels in Cardinal table grape skin by low temperature and high CO_2_. J. Plant. Physiol. 179, 1–11. 10.1016/j.jplph.2015.02.007 25817412

[B46] Nunes M. C. N. Morais A. M. M. B. Brecht J. K. Sargent S. A. (2002). Fruit maturity and storage temperature influence response of strawberries to controlled atmospheres. J. Am. Soc. Hort. Sci. 127, 836–842.

[B47] Pavez L. Hödar C. Olivares F. González M. Cambiazo V. (2013). Effects of postharvest treatments on gene expression in *Prunus persica* fruit: normal and altered ripening. Postharvest Biol. Technol. 75, 125–134. 10.1016/j.postharvbio.2012.08.002

[B48] Plotkin J. B. (2010). Transcriptional regulation is only half the story. Mol. Syst. Biol. 6:406. 10.1038/msb.2010.63 20739928 PMC2950086

[B49] Ponce-Valadez M. Moore S. Giovannoni J. J. Gan S. Watkins C. B. (2009). Differential fruit gene expression in two strawberry cultivars in response to elevated CO_2_ during storage revealed by a heterologous fruit microarray approach. Postharvest Biol. Technol. 51, 131–140. 10.1016/j.postharvbio.2008.08.001

[B50] Qian C. He Z. Zhao Y. Mi H. Chen X. Mao L. (2013). Maturity-dependent chilling tolerance regulated by the antioxidative capacity in postharvest cucumber (*Cucumis sativus* L.) fruit. J. Sci. Food Agric. 93, 626–633. 10.1002/jsfa.5858 22936358

[B51] Romero I. Fernandez-Caballero C. Sanchez-Ballesta M. T. Escribano M. I. Merodio C. (2009). Influence of the stage of ripeness on phenolic metabolism and antioxidant activity in table grapes exposed to different CO_2_ treatments. Postharvest Biol. Technol. 54, 118–121. 10.1016/j.postharvbio.2009.05.002

[B52] Romero I. Sanchez-Ballesta M. T. Maldonado R. Escribano M. I. Merodio C. (2006). Expression of a class I chitinase and β-1,3-glucanase genes and postharvest fungal decay control of table grapes by high CO_2_ pretreatment. Postharvest Biol. Technol. 41, 9–15. 10.1016/j.postharvbio.2006.03.001

[B53] Rosales R. Fernandez-Caballero C. Romero I. Escribano M. I. Merodio C. Sanchez-Ballesta M. T. (2013). Molecular analysis of the improvement in rachis quality by high CO_2_ levels in table grapes stored at low temperature. Postharvest Biol. Technol. 77, 50–58. 10.1016/j.postharvbio.2012.10.009

[B54] Saeed A. I. Sharov V. White J. Li J. Liang W. Bhagabati N.. (2003). TM4: a free, open-source system for microarray data management and analysis. Biotechniques 34, 374–378. 12613259 10.2144/03342mt01

[B55] Salvador A. Arnal L. Monterde A. Martínez-Jávega J. M. (2005). Influence of ripening stage at harvest on chilling injury symptoms of persimmon cv. Rojo Brillante stored at different temperatures. Food Sci. Technol. Int. 11, 359–365. 10.1177/1082013205057941

[B56] Sanchez-Ballesta M. T. Jiménez J. B. Romero I. Orea J. M. Maldonado R. Ureña A. G. (2006). Effect of high CO_2_ pretreatment on quality, fungal decay and molecular regulation of stilbene phytoalexin biosynthesis in stored table grapes. Postharvest Biol. Technol. 42, 209–216. 10.1016/j.postharvbio.2006.07.002

[B57] Sanchez-Bel P. Egea I. Sanchez-Ballesta M. T. Sevillano L. Bolarin M. C. Flores F. B. (2012). Proteome changes in tomato fruits prior to visible symptoms of chilling injury are linked to defensive mechanisms, uncoupling of photosynthetic processes and protein degradation machinery. Plant Cell Physiol. 53, 470–484. 10.1093/pcp/pcr191 22227396

[B58] Sanhueza D. Vizoso P. Balic I. Campos-Vargas R. Meneses C. (2015). Transcriptomic analysis of fruit stored under cold conditions using controlled atmosphere in *Prunus persica* cv. “Red Pearl.” Front. Plant Sci. 6:788. 10.3389/fpls.2015.00788 26483806 PMC4586424

[B59] Secco D. Wang C. Arpat B. A. Wang Z. Poirier Y. Tyerman S. D.. (2012). The emerging importance of the SPX domain-containing proteins in phosphate homeostasis. New Phytol. 193, 842–851. 10.1111/j.1469-8137.2011.04002.x 22403821

[B60] Shin Y. Ryu J. A. Liu R. H. Nock J. F. Watkins C. B. (2008). Harvest maturity, storage temperature and relative humidity affect fruit quality, antioxidant contents and activity, and inhibition of cell proliferation of strawberry fruit. Postharvest Biol. Technol. 49, 201–209. 10.1016/j.postharvbio.2008.02.008

[B61] Terry L. A. Crisosto C. H. Forney C. F. (2009). Small fruit and berries in Modified and Controlled Atmospheres for the Storage, Transportation, and Packaging of Horticultural Commodities, ed Yahia E. M. (Boca Raton, FL: CRC Press), 363–395.

[B62] To T. K. Nakaminami K. Kim J. M. Morosawa T. Ishida J. Tanaka M.. (2011). *Arabidopsis HDA6* is required for freezing tolerance. Biochem. Biophys. Res. Commun. 406, 414–419. 10.1016/j.bbrc.2011.02.058 21329671

[B63] UNECE (2010). Standard FFV-19 Concerning the Marketing and Commercial Quality Control of Table Grapes. Available online at: https://www.unece.org/fileadmin/DAM/trade/agr/standard/fresh/FFV-Std/English/19TableGrapes.pdf

[B64] Vitulo N. Forcato C. Carpinelli E. C. Telatin A. Campagna D. D'Angelo M.. (2014). A deep survey of alternative splicing in grape reveals changes in the splicing machinery related to tissue, stress condition and genotype. BMC Plant Biol. 14:99. 10.1186/1471-2229-14-99 24739459 PMC4108029

[B65] Wang L. Zhu W. Fang L. Sun X. Su L. Liang Z.. (2014). Genome-wide identification of WRKY family genes and their response to cold stress in *Vitis vinifera*. BMC Plant Biol. 14:103. 10.1186/1471-2229-14-103 24755338 PMC4021059

[B66] Xin H. Zhu W. Wang L. Xiang Y. Fang L. Li J.. (2013). Genome wide transcriptional profile analysis of *Vitis amurensis* and *Vitis vinifera* in response to cold stress. PLoS ONE 8:58740. 10.1371/journal.pone.0058740 23516547 PMC3596283

[B67] Xuan J. Song Y. Zhang H. Liu J. Guo Z. Hua Y. (2013). Comparative proteomic analysis of the stolon cold stress response between the C4 perennial grass species *Zoysia japonica* and *Zoysia metrella*. PLoS ONE 26:75705 10.1371/journal.pone.0075705 PMC3784457 24086619

[B68] Yang L. A. Ji W. Zhu Y. M. Gao P. Li Y. Cai H.. (2010). *GsCBRLK*, a calcium/calmodulin-binding receptor-like kinase, is a positive regulator of plant tolerance to salt and ABA stress. J. Exp. Bot. 61, 2519–2533. 10.1093/jxb/erq084 20400529

[B69] Yoshida S. Hotsubo K. Kawamura Y. Murai M. Arakawa K. Takezawa D. (1999). Alterations of intracellular pH in response to low temperature stress. J. Plant Res. 112, 225–236. 10.1007/PL00013879

[B70] Yun Z. Jin S. Ding Y. Wang Z. Gao H. Pan Z.. (2012). Comparative transcriptomics and proteomics analysis of citrus fruit, to improve understanding of the effect of low temperature on maintaining fruit quality during lengthy post-harvest storage. J. Exp. Bot. 63, 2873–2893. 10.1093/jxb/err390 22323274 PMC3350911

[B71] Zeng Y. Yang T. (2002). RNA isolation from highly viscous samples rich in polyphenols and polysaccharides. Plant Mol. Biol. Rep. 20, 417 10.1007/BF02772130

[B72] Zhang C. F. Din Z. Xu X. Wang Q. Qin G. Tian S. (2010). Crucial roles of membrane stability and its related proteins in the tolerance of peach fruit to chilling injury. Amino Acids 34, 181–194. 10.1007/s00726-009-0397-6 20091071

[B73] Zhao L. Liu F. Xu W. Di C. Zhou S. Xue Y.. (2009a). Increased expression of *OsSPX1* enhances cold /subfreezing tolerance in tobacco and *Arabidopsis thaliana*. Plant Biotechnol. J. 7, 550–561. 10.1111/j.1467-7652.2009.00423.x 19508276

[B74] Zhao Z. Cao J. Jiang W. Gu Y. Zhao Y. (2009b). Maturity-related chilling tolerance in mango fruit and the antioxidant capacity involved. J. Sci. Food Agric. 89, 304–309. 10.1002/jsfa.3443

[B75] Zhu J. Jeong J. C. Zhu Y. Sokolchik I. Miyazaki S. Zhu J. K.. (2008). Involvement of *Arabidopsis* HOS15 in histone deacetylation and cold tolerance. Proc. Natl. Acad. Sci. U.S.A. 105, 4945–4950. 10.1073/pnas.0801029105 18356294 PMC2290775

