# Supplementary material for: Low Temperature and Short-Term High-CO2 Treatment in Postharvest Storage of Table Grapes at Two Maturity Stages: Effects on Transcriptome Profiling
Source: Front Plant Sci. 2016 Jul 13;7:1020. doi: 10.3389/fpls.2016.01020 (PMC4942463; doi:10.3389/fpls.2016.01020)
Supplement: Supplementary file 7 [file Image1.PDF]

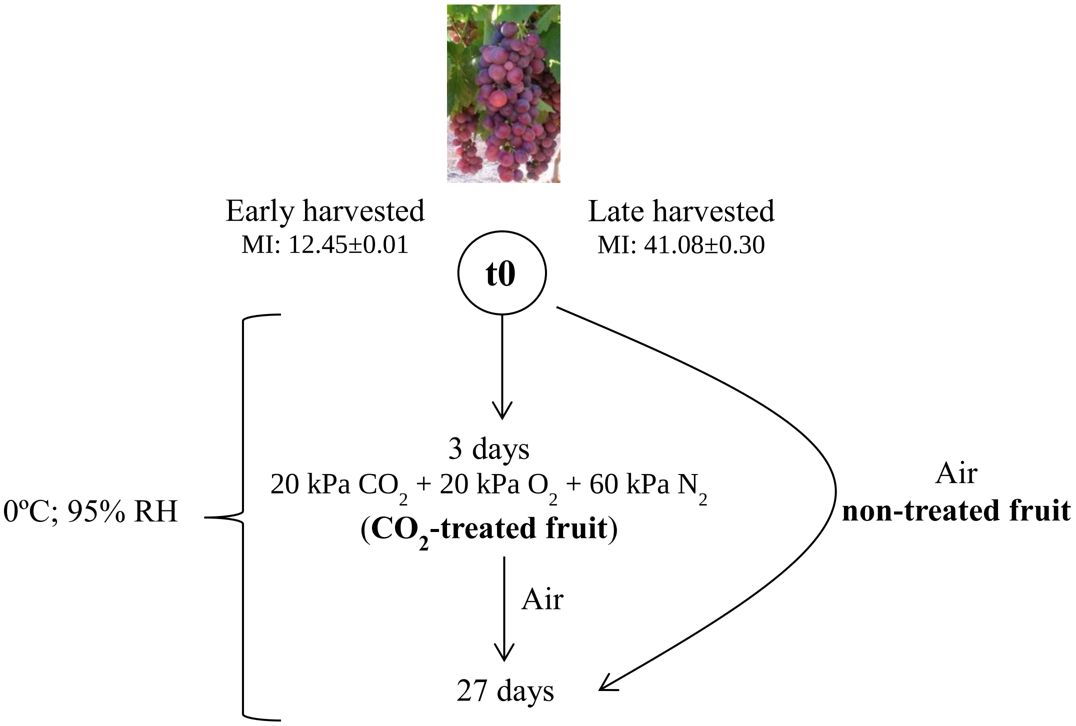

Supplementary Figure S1. Scheme summarizing the experimental set-up. Table grapes (*Vitis vinifera* L. cv. Cardinal) harvested in Sevilla (Spain) at two maturity stages were transported to the laboratory in Madrid (Spain) in a refrigerated van. Bunches free from physical and pathological defects (t0) were randomly divided into two lots and stored up to 27 days at 0±0.5ºC and 95% RH in two sealed neoprene containers of 1 m^3 capacity. One lot was kept under normal atmosphere (non-treated fruit) and the other one was treated with high CO_2 levels (CO_2-treated fruit) during 3 days. Subsequently, CO_2-treated grapes were transferred to air under the same conditions as the non-treated fruit until the end of the storage period. Transcriptomic microarray analysis was conducted on RNA samples from the skin of early- and late-harvested table grapes at time 0 and after 3 days of storage at 0ºC under air or high CO_2 conditions.
